# Spatial mismatch analysis among hotspots of alien plant species, road and railway networks in Germany and Austria

**DOI:** 10.1371/journal.pone.0183691

**Published:** 2017-08-22

**Authors:** Yanina Benedetti, Federico Morelli

**Affiliations:** Department of Applied Geoinformatics and Spatial Planning Faculty of Environmental Sciences, Czech University of Life Sciences Prague, Prague, Czech Republic; Universidade de Lisboa Instituto Superior de Agronomia, PORTUGAL

## Abstract

Road and railway networks are pervasive elements of all environments, which have expanded intensively over the last century in all European countries. These transportation infrastructures have major impacts on the surrounding landscape, representing a threat to biodiversity. Roadsides and railways may function as corridors for dispersal of alien species in fragmented landscapes. However, only few studies have explored the spread of invasive species in relationship to transport network at large spatial scales. We performed a spatial mismatch analysis, based on a spatially explicit correlation test, to investigate whether alien plant species hotspots in Germany and Austria correspond to areas of high density of roads and railways. We tested this independently of the effects of dominant environments in each spatial unit, in order to focus just on the correlation between occurrence of alien species and density of linear transportation infrastructures. We found a significant spatial association between alien plant species hotspots distribution and roads and railways density in both countries. As expected, anthropogenic landscapes, such as urban areas, harbored more alien plant species, followed by water bodies. However, our findings suggested that the distribution of neobiota is strongest correlated to road/railways density than to land use composition. This study provides new evidence, from a transnational scale, that alien plants can use roadsides and rail networks as colonization corridors. Furthermore, our approach contributes to the understanding on alien plant species distribution at large spatial scale by the combination with spatial modeling procedures.

## Introduction

During the last century, linear human related infrastructures such as roads and railways have become a conspicuous part of the anthropogenic landscape [1,2]. In Europe, roads have been constructed for more than 2000 years, but expansion of this communication system is accelerating, constituting, together with land-use, climate change, pollution, and other infrastructural developments, a main driver of biodiversity decline [3,4]. Currently there are about 35,500,000 kms of roads globally, according to data provided by the World Development Index of World Bank, World Road Statistics of the International Road Federation and of World Factbook of Central Intelligence Agency on the World [5].

Roads threaten biodiversity in at least three different ways: 1) fragment and modify landscapes directly, 2) modify landscapes indirectly by promoting urbanization and, 3) modify landscapes by facilitating the movement of people and goods, thereby increases the risk of biological invasions [6]. Then, ecological disturbances caused by human activity related to roads and railways, contribute to the spread of non-native species when their construction involves the movement of soil contaminated with propagules of invasive species [7]. Bacaro et al. [8], described three main mechanisms for how roads increase dispersal of propagules of alien species: 1) they are a source of disturbance, creating new environmental conditions that are suitable to ruderal and pioneer species, 2) they facilitate the dispersal of propagules via air movement associated with the transit of vehicles, and 3) they facilitate colonization by alien species by suppressing the growth or removing stands of native species [8,9].

Roads and railways therefore serve as dispersal corridors for many plant species, particularly invasive ones [8,10–16]. These movements of species can cause significant changes in affected ecosystems, because invasive species that compete with native ones can alter native communities in all kind of landscapes [16–18]. Indeed, invasive species constitute a major threat to biological diversity all around the world [19,20].

Abiotic and biotic variables together with dispersal mechanism strongly influence spatial pattern and invasion rate of invasive species [21–25]. The close association between plant invasions with roads and railways has long been established and repeatedly documented in several studies [8,10–12,15,26–37]. However, most of these studies were focused at local spatial scale, using detailed data collected by sample plots or linear transects [8,15,31,37]. Very few research were focused on railways networks [32,38–40], and—most important—only few studies have explored the spread of invasive species at large spatial scales [41,42], but these works were not focused on alien plants distribution in relationship to the effects of both road/railways networks and land use composition.

Understanding the factors which facilitate and drive the spread of invasive species is necessary for developing appropriate strategies for preventing and limiting ecological invasions. Considering that: a) road and railways networks are among the most pervasive landscape features accompanying the urbanization process; b) most of the studies which verifyied that these infrastructures provide a vector of diffusion for neobiota were performed at local spatial scale; and c) plant invaders are more frequently found in highly disturbed anthropogenic habitats; we can expect a positive spatially association between the road/railways density and number of aliens plant species at large spatial scale. Thus, an eco-informatic approach, based on a large set of spatially explicit data focusing the pattern of alien plants, can provide new insights on invasion ecology.

In according to this, the aim of our study is to provide a spatially explicit correlation analysis at large spatial scale, in order to assess the congruence between hotspots of invasive plant species and density of linear transports elements in two countries of central Europe. Furthermore, by modeling, to assess the relative role of road/railway density and land use composition on the distribution of alien plant species. Then, our approach can contribute to improve the knowledge on alien plant distribution by the combination with spatial modeling, to obtain more accurate predictive frameworks at large or regional spatial scale.

## Methods

### Alien plant invasion hotspots data source

We used the available records of hotspots of alien vascular plant species using the database of “Actual and potential future alien plant invasion hotspots under two emissions scenarios” [43], a report available in https://www.eea.europa.eu/data-and-maps. This report provided a dataset with 13,373 records, with number of alien plant species per spatial unit, quantified following the methods of Kleinbauer et al. [44]. Each record was assigned to a grid cell 5.5 km x 5.5 km (5 · 3 geographic minutes, approximately 30.25 km^2^) of the Floristic Mapping Project of Central Europe (FMA; Niklfeld, 1998). Each record presents the frequencies of 30 invasive alien vascular plant species in Austria and Germany (see detailed explanation on http://www.eea.europa.eu/data-and-maps/figures/actual-and-potentialfuture-alien). In this study, each FMA square was defined as the spatial unit for the further statistical and spatial analysis.

### Roads and railways networks and spatial density

The maps were generated with GIS soſtware (ArcGIS 10.1) [45] with geographical background using data available under the Open Database Licence (“OpenStreetMap and contributors”; cartography licensed as CC BY-SA), http://www.openstreetmap.org/copyright. The following layers were used: road network and railways network from Germany and Austria. Roads term included both motorways and residential roads. The vector data is derived from CORINE land-cover (25-m resolution) [46]. Road density and railway density were calculated using the command “line density” from Spatial Analyst in ArcGIS 10.1 [45]. The line density tool calculates the density of linear features in the neighborhood of each output raster cell, as the units of length per unit of area [47]. In this study, the density of linear structures (road and railways) was computed as the total length in kms per each km^2^ in each spatial unit.

### Classification of spatial units on a dominant environment

Each spatial unit was classified on the basis of the percentages of the different land uses types within each square. Land-use types considered here were based on the CORINE land-cover vector data derived from 25-m resolution satellite data from 2006. CORINE is a national georeferenced land-cover database available for the European Union, based on satellite digital images [46]. The CORINE provides classified spatial land cover data in GIS format organized hierarchically in three-level CORINE nomenclature [48], and has been used to define the different European land covers. The CORINE system includes 44 land cover classes. Land-use categories taken from CORINE Land Cover (CLC) were grouped to obtain the 5 land-use types used in this study (i.e. urban, agricultural, forest and seminatural environments, waterbodies and coast area or wetland). The percentage of each land-use type was calculated by ArcGIS 10.1 software [45], using “intersect operator” between the grids (spatial units) and the CLC map for both countries, obtaining a crosstab matrix. Units were classified in terms of dominant environment in each category when the main land use was >60% [49], with the exception of the category “urban”, which had a lower threshold of >30%. Units with mixed composition, where none of land-use types had at least 60%, were classified as mixed environments.

### Statistical analyses

A preliminary exploration of the correlation among variables was performed using the Pearson correlation coefficient [50] (S1 Fig). The comparison between the spatial pattern of alien plant species occurrence and density of roads and railways was initially explored using spatially explicit tests (which consider the correlations in contiguous areas). The spatial associations were tested using Mantel tests [51]. The statistic r_M_ varies between −1 and +1 and behaves like a correlation coefficient [52]; it evaluates the similarity between two distance matrices [53]. Mantel tests were also used to check for spatial autocorrelation of data [54], comparing the geographic distance matrix and the matrix of differences in number alien plant species among spatial units. Monte Carlo permutations with 9999 randomizations were used to test for significance [55].

The relationship between hotspots of alien plant species and road and railway density in each spatial unit was examined using Generalized Linear Mixed Models (GLMMs), with the package ‘lme4’ [56,57]. Number of alien plant species was modeled as response variable and road and railways density as fixed factors [58], while the interaction between country and dominant environment was included as random factor to control for possible consistent differences among countries and environments (model 1). Variance inflation factor (VIF) was calculated to examine whether there is no risk of multicollinearity between predictors (road and railway density), but the value was 1.46 (< 2) suggesting that there was no collinearity issues in our dataset [59]. VIF was estimated using ‘fmsb’ package [60]. Model was fitted assuming a Poisson distribution after having explored the distribution of variables using the package ‘MASS’ [61]. The confidence intervals for the significant variables were calculated using the Wald method from the package ‘MASS’ [61].

In order to focus separately the role of road / railways density and land use composition on the hotspots of neobiota, we adopted a double approach. First, a new series of Generalized Linear Models (GLM) was ran using the number of alien plant species as response variable, introducing road density and country as predictors (model 2), and then the land use composition (measured as the percentage of each land use type) and country as predictors (model 3). Country was added in the model to explore if some variation in neobiota occurrence is due to intrinsic differences of each country. For example, considering that Germany is biggest country than Austria, we can expect also more linear structures in terms of absolute values, and then a potential increase in number of neobiota. Moreover, these differences can also mirror the higher population density in Germany [62], and a slightly great coverage of land uses potentially associated positively to alien plant species (as urban or water bodies). Finally, because the occurrence of alien plant species is negatively correlated to the gradient of elevation [63], we can expect an overall lower number of alien species in Austria than in Germany, since average elevation for Germany is lower than Austria [62]. In order to avoid redundancy, only road density was modeled in model 2, because railways density is strongly spatially correlated with road network (S1 Fig). Road density and land use composition were modeled separately, in order to avoid multicollinearity related to a differential distribution of road networks in each type of land use. Akaike’s Information Criterion (AIC) was used to determine the performance of best models explaining variation in the data [64]. The assessment of the variance explained for the models was based on a comparison between observed and predicted values from the fitted models [65]. For the computation of R square in Generalized Linear Mixed models (GLMM) we followed the method explained by Nakagawa and Schielzeth [66].

Second, a variation partitioning by partial regression analysis was used to isolate the proportion of the variation on neobiota explained by each of the two sets of factors exclusively (road / railways density and land use composition), and the proportions attributable to interactions between factors [67,68]. To test whether explanatory variables account for a significant variance, we used function ‘rda’ to test for fractions. Variation partitioning was performed using the ‘vegan’ package for R [55].

All statistical tests were performed with R software [69].

## Results

**Fig 1:**
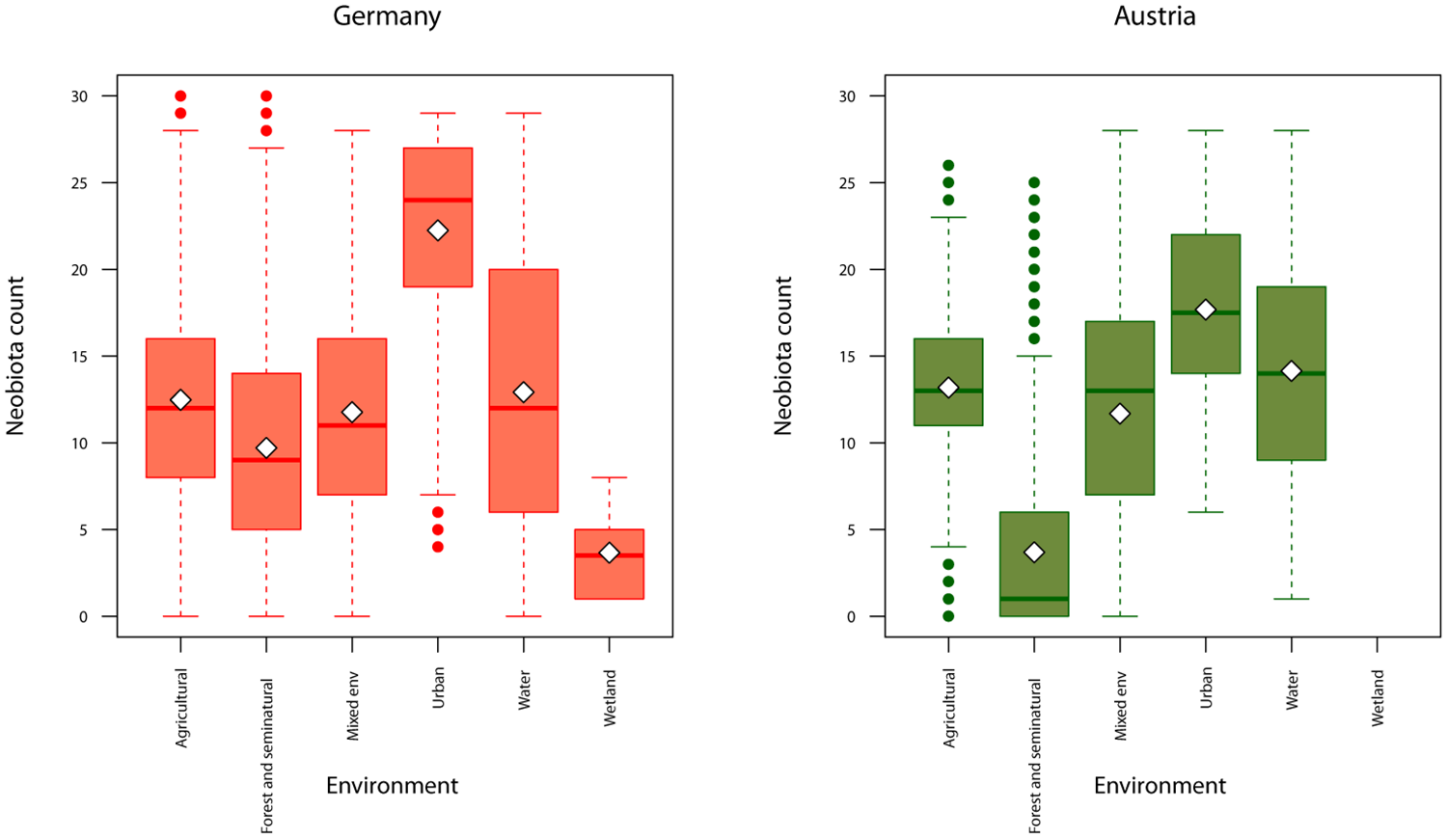
Number of alien plant species in relation to the dominant environment of each spatial unit. This elaboration is based on the intersection between data of alien plant species hotspots [43] and the land use composition in each 5.5 km x 5.5 km spatial unit extracted from CORINE land cover for Germany and Austria [48]. The box plots show medians, quartiles, 5- and 95-percentiles and extreme values.

The maximum number of invasive plant species per spatial unit (30.25 km^2^) was 28 for Austria and 30 for Germany. On average, grid units contained 6.86 species in Austria and 12.09 in Germany. In both countries, the hotspots of alien plant species were more related to anthropogenic landscapes, such as urban habitat types (Fig 1). The land use types with the next highest number of alien plant species were water bodies and agricultural. The forest and seminatural areas and wetlands contained the fewest alien plant species (Fig 1).

We found a maximum value of 12.77 km of road per km^2^ for Austria and 11.68 km per km^2^ for Germany, with average values of 1.07 km per km^2^ for Austria and 1.06 km per km^2^ for Germany. The maximum value for railways density was 5.48 km per per km^2^ in Austria and 7.33 km per km^2^ in Germany, with average values of 0.28 km per km^2^ in Austria to 0.36 km per km^2^ in Germany.

**Fig 2:**
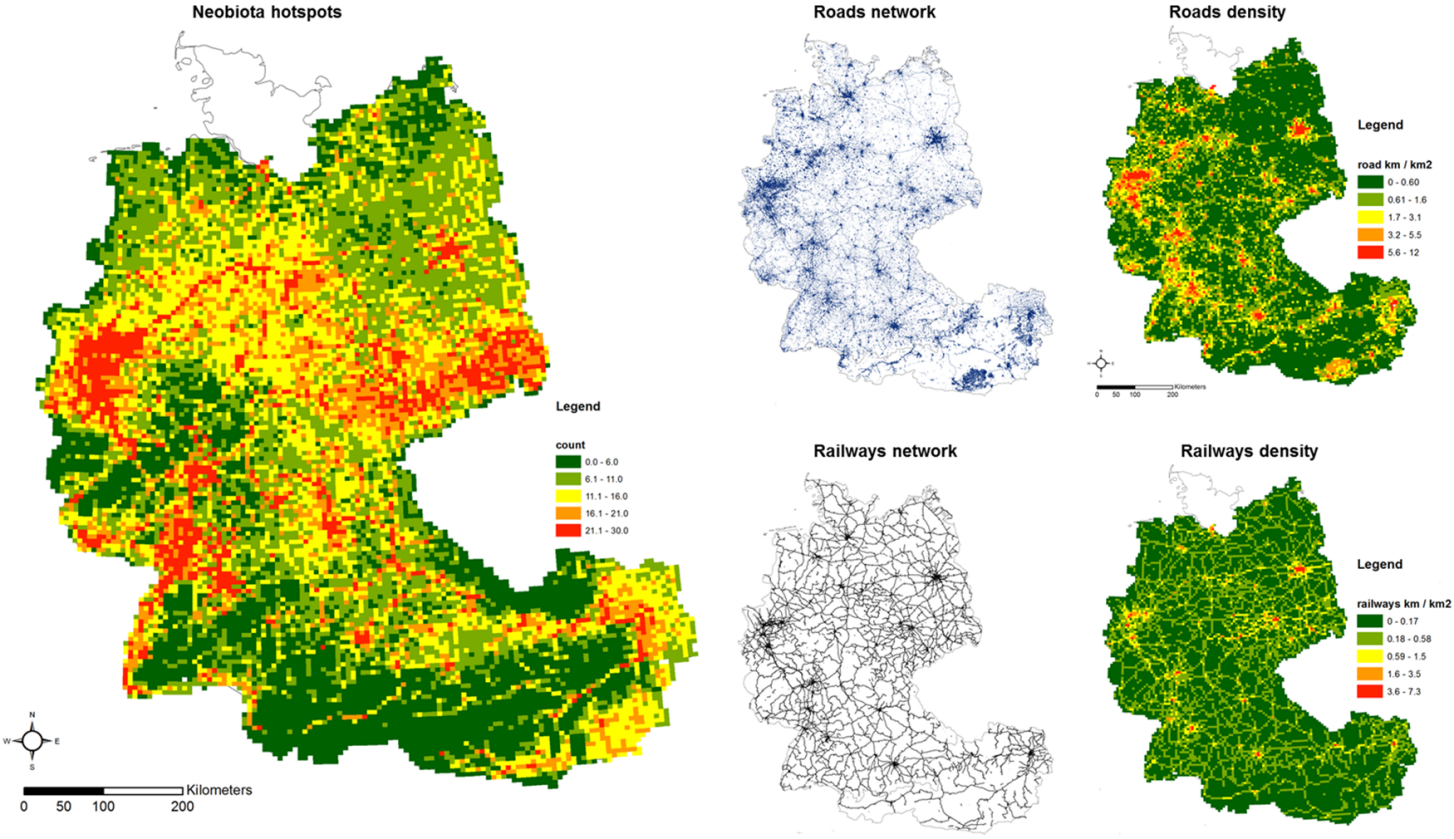
Alien plant species hotspot distribution, road and railway networks and density distribution in Germany and Austria. All maps were generated with GIS soſtware (ArcGIS 10.1) [45] with geographical background using data available under the Open Database Licence (“OpenStreetMap and contributors”; cartography licensed as CC BY-SA) http://www.openstreetmap.org/copyright, and the map of alien plant species hotspots was based on free available data from http://www.eea.europa.eu/data-and-maps/figures/actual-and-potential-future-alien. All values were calculated by intersection in each spatial unit 5.5 km x 5.5 km.

Spatial grid units were treated as statistically independent observations because the values of spatial autocorrelation for the number of alien plant species calculated by comparing the geographic distance matrix with that of dissimilarity in number of alien plant species was weak and not significant (r_M_ = 0.038, permutations = 9999, p > 0.05) [70]. Spatial pattern of alien plant species was congruent with spatial pattern of road network (r_M_ = 0.330, permutations = 9999, p < 0.01) and railways network (r_M_ = 0.343, permutations = 9999, p < 0.01) in Germany and Austria, indicating that grid units that differed more in number of alien plant species also differed more in density of linear transport features (Fig 2).

**Fig 3:**
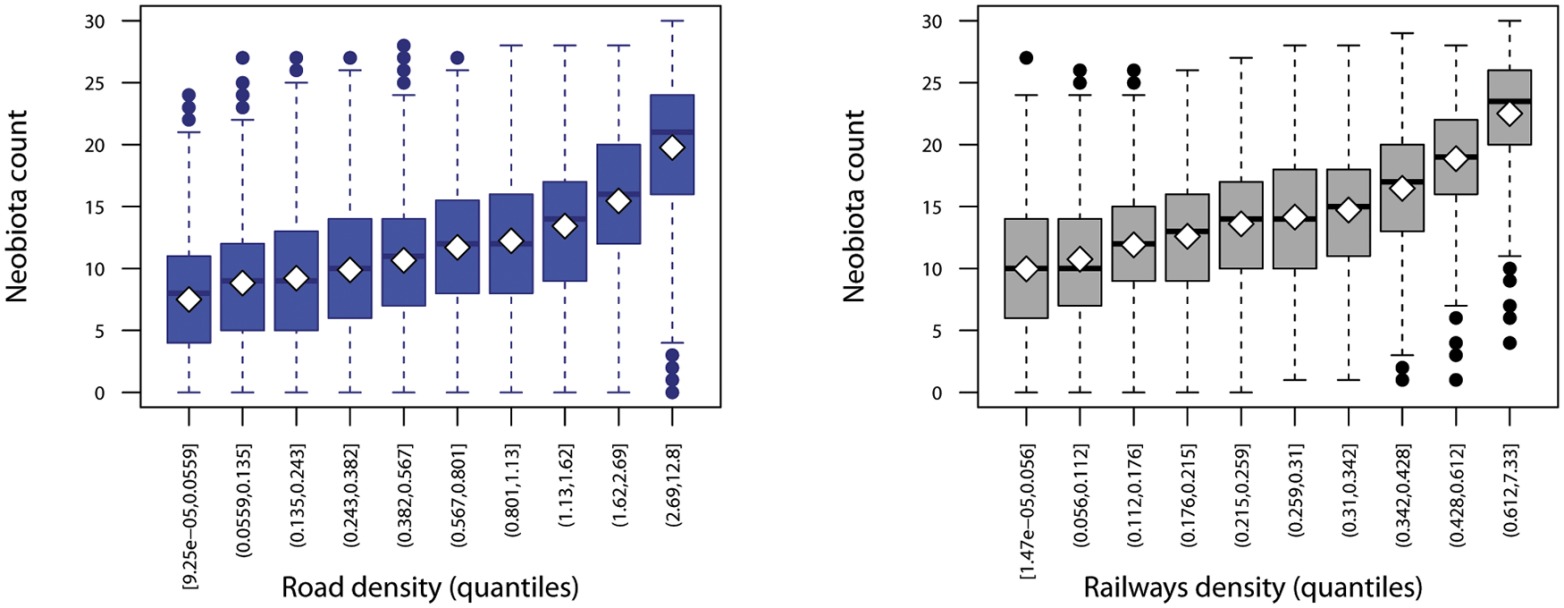
Number of alien plant species in relation to road density and railway density. These relationships were estimated as km of linear structures per km^2^, classified in quantiles in each 5.5 km x 5.5 km spatial unit in Germany and Austria. The box plots show medians, quartiles, 5- and 95-percentiles and extreme values.

The mixed models evaluate the association between number of alien plant species and road density and railways density, independently from country and dominant environment. The results confirmed that grid units with higher road density and with higher railways density (model 1) contained more number alien plant species (Table 1, Fig 3). The variance explained by the model based on road/railways density was 45%.

**Table 1:** Results of GLMM for best model relating number of alien plant species to road density and railway density for each spatial unit in Germany and Austria. The interaction between country and dominant environment was added as random factor in the model (11 groups). The table shows estimates, 95% confidence intervals (CI), SE, Z and p values.

Predictors / model	Estimate	CI	SE	Z	p
Model 1: road and railway density					
Intercept	2.444	2.071 / 2.374	0.042	57.37	<2e-16
Road density	0.085	0.081 / 0.089	0.002	40.63	<2e-16
Railways density	0.095	0.084 / 0.107	0.006	16.09	<2e-16

The last two models, including separately road density and land use composition on each spatial unit as potential predictors, showed a positive association between number of alien plant species and road density and country Germany (model 2, Table 2). The strongest positive association between the number of alien plant species and land use categories was found for urban environments, while the strongest negative associations were found for wetlands and forest and semi-natural areas (model 3, Table 2). Finally, comparing the performance of the last two models, the first one (road density model) was largely superior, presenting the lower AIC (Table 3). The variance explained by the model based on land use composition was 19%.

**Table 2:** Results of GLM for best models relating number of alien plant species separately to road density and country (model 2) and to dominant environment based on land use composition and country (model 3) for each spatial unit in Germany and Austria. Only significant variables are shown in table. The table shows estimates, 95% confidence intervals (CI), SE, Z and p values.

Predictors / model	Estimate	CI	SE	Z	p
**Model 2: road density**					
Intercept	2.002	1.987 / 2.017	0.008	258.44	<2e-16
Road density	0.149	0.147 / 0.152	0.001	107.40	<2e-16
Country: Germany	0.335	0.319 / 0.351	0.008	41.98	<2e-16
**Model 3: land use**					
Intercept	2.207	2.191 / 2.224	0.008	262.24	<2e-16
Forest and semi-natural	-0.482	-0.497 / -0.467	0.007	-64.71	<2e-16
Mixed	-0.022	-0.039 / -0.005	0.009	-2.48	0.013
Urban	0.559	0.531 / 0.588	0.014	38.74	<2e-16
Wetland	-1.251	-1.700 / -0.860	0.213	-5.86	4.4e-09
Water	0.073	0.049 / 0.097	0.012	6.07	<1.2e-09
Country: Germany	0.343	0.327 / 0.359	0.008	41.72	<2e-16

**Table 3:** List of GLMs performed in this study, relating number of alien plant species separately to road density (model 2) and to dominant environment based on land use composition (model 3) for each spatial unit in Germany and Austria. The choice of the best model is based on Akaike's information criterion (AIC) in the package AICcmodavg from R (Mazerolle, 2016).

Model	No. variables	AIC	ΔAIC
Road density	2	86775.6	0
Environment (land use)	6	107535.2	20759.7
Null model	1	120456.3	33680.7

**Fig 4:**
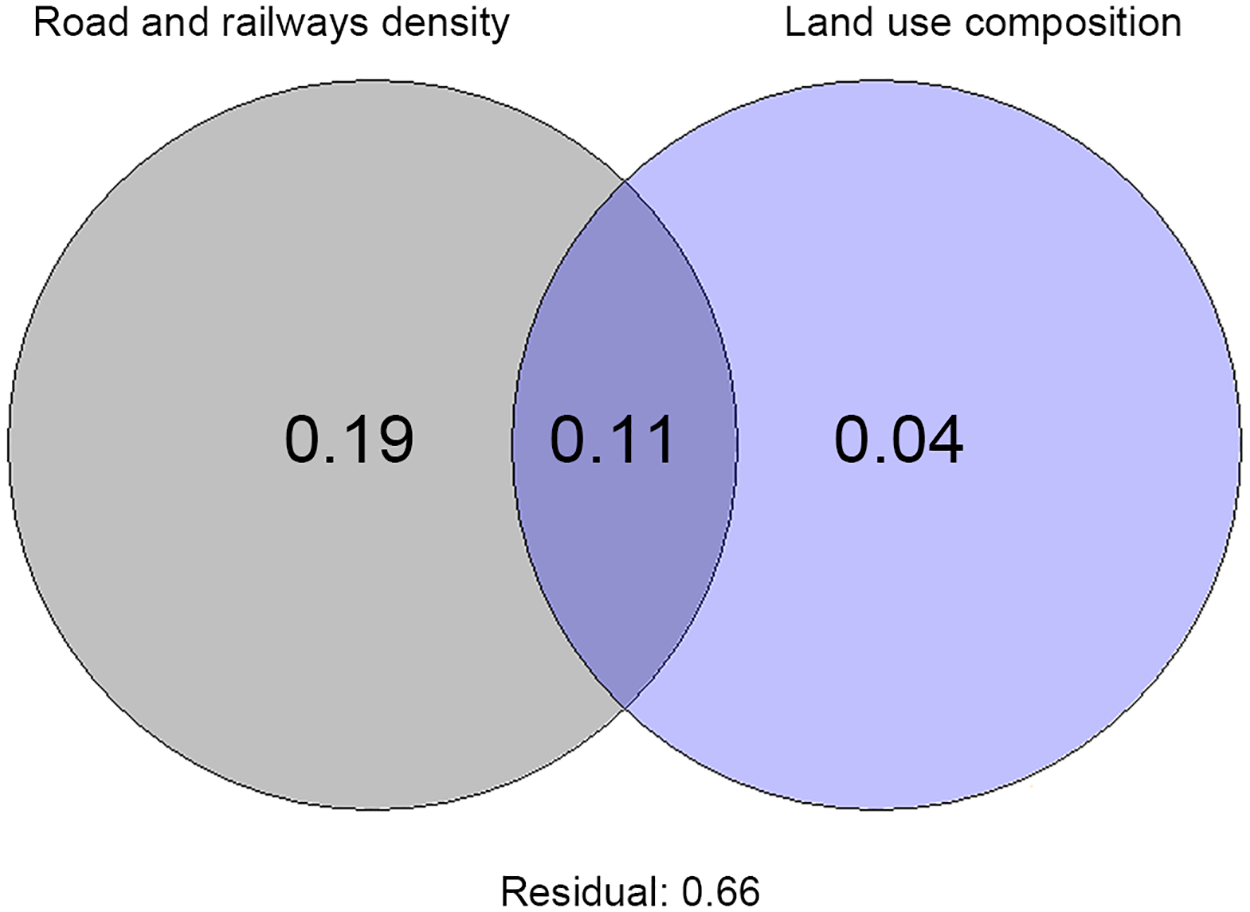
Venn diagram showing the results of variation partitioning analysis on number of alien plant species in relation to road and railways density and the land use composition in each spatial unit. The diagrams represent the adjusted percentages of unique contribution of road and railways density, and dominant environment based on land use composition in Germany and Austria. The fraction between two overlapped circles represents the variation explained between the components while the residuals are the variation left unexplained by the canonical model. The fractions of variation displayed in the diagram are computed from adjusted r^2^. Both unique contributions were statistically significant (*).

The results of variation partitioning analysis provided additional confirmation that number of alien plant species is significantly correlated with road/railways density and land use composition. However, the large effect was found for road/railways density, followed by the intersection between road/railways density and land sue composition, and then land use composition in each spatial unit (Fig 4).

## Discussion

Historical and current interactions between abiotic and biotic variables, including anthropogenic influences to natural communities and landscapes, determines invasion rate and distribution pattern of all alien species throughout the world [23–25,39,71]. Invasive species are often physiologically plastic [72], which permits them to colonize new environments, particularly disturbed ones modified by humans. Good knowledge of their ecological aptitude and dispersal strategies are essential for developing and deploying relevant, effective strategies to prevent and minimize or to control invasion [73,74].

Only spatially explicit information based on large dataset can be used to elaborate accurate macroecological patterns of plant invasions, suitable to predict or identify the areas of potential risk of invasions in future scenarios, as well as to elaborate adequate conservation strategies [75]. In this regard, the main innovative aspects of our study are: a) the use of spatially explicit analysis; b) the focus on associations among alien plant distribution, road/railways density and land use composition at a large spatial scale; and c) the potentialities to create predictive models at large spatial scale offered by the modeling approach.

In this study, we provide new evidence highlighting the spatial congruence between the pattern of hotspots of alien plant species and the pattern of road and railway density at large spatial scales in two European countries. This approach remains descriptive, and cannot truly demonstrate causality between alien plant species and modern transportation infrastructures. However, other studies provide additional compelling evidence for how roads and railways are instrumental in spreading of invasive species (see for example: [7,11,12,76]). Our study therefore confirms these ideas but at a much larger geographical scale, by applying a modeling approach.

We found that while the average values on human-related infrastructures (roads and railways density) were similar between countries, the average values of neobiota was higher in Germany. This difference could be partially due to differences on the gradient of elevation between both countries [63].

The distribution and expansion of alien plants depends on roads as a main driver, but is also related to the land use composition around the roads [36]. The urban areas can be considered at higher risk of invasion, mainly because are habitats characterized by large fluctuations of resources availability, and the invasive species are more adaptable to strongly disturbed environments [75]. We, similarly, found more alien plant species in sampling units that had higher representation of urban habitats and of water bodies, over and above the effect of road and rail networks. On the other hand, we found the lower values of invasive plant species in wetlands [76] and forest areas. Similar results have been observed in other studies, evidencing that the frequency of alien plant species decline in forest habitat [39,77]. Additionaly, our findings provided a demonstration that the distribution of neobiota is strongest correlated to road/railways density than to land use composition. This result was emphasized by comparing both the explained variance and AIC of best models performed separately for both predictors, as well as by the direct comparison of the isolated proportion of variation on neobiota data explained by each of these two sets of factors exclusively.

Bacaro et al. [8], highlighted the major role of road edges as well as the distance from the road side [8,78] in determining plant species richness distributions. Much of this effect is due to the number of alien species increasing close to roads, while less disturbed areas (away from roads) are characterized by fewer alien species [9]. Several studies show that regular road maintenance can spread alien plant species, for example on the maintenance machinery or the footwear of road maintenance workers. Furthermore alien species may establish particularly well in the disturbed soil or cleared areas associated with road maintenance work [79,80].

The importance of transport networks on alien plant species is particularly important, since the accumulation of alien species across all taxonomic groups shows no sign of saturation, worldwide [81]. Invasive species continue to arrive and establish, and the accelerating increase in transportation networks will only facilitate this process, emphasizing the importance for conservation of roadless areas [82]. Knowing the most threatened habitat types and situations can help direct oversight efforts at local and regional scales. This can therefore help to set in place adequate measures for early detection and more efficient control measures for invasive species.

## Supporting information

S1 Fig Correlation among road density, railways density and land use composition in each spatial unit for Germany and Austria. The diagonal shows the name of variables using the following codes: URB, urban; AGRI, agricultural, FOR, forest and semi-natural areas; WET, wetlands; WAT, waterbodies; ROAD, road density; RAIL, railways density. The squares below the diagonal show the bivariate plots and the squares above the diagonal the corresponding correlation coefficients, with level of significance indicated by symbols (.,*,**).
(DOC)
