# Supplementary material for: Spatial mismatch analysis among hotspots of alien plant species, road and railway networks in Germany and Austria
Source: PLoS One. 2017 Aug 22;12(8):e0183691. doi: 10.1371/journal.pone.0183691 (PMC5567654; doi:10.1371/journal.pone.0183691)
Supplement: S1 Fig — The diagonal shows the name of variables using the following codes: URB, urban; AGRI, agricultural, FOR, forest and semi-natural areas; WET, wetlands; WAT, waterbodies; ROAD, road density; RAIL, railways density. The squares below the diagonal show the bivariate plots and the squares above the diagonal the corresponding correlation coefficients, with level of significance indicated by symbols (.,*,**). (DOC) [file pone.0183691.s001.doc]

**Supporting information**

**S1 Fig Correlation among road density, railways density and land use composition in each spatial unit for Germany and Austria.** The diagonal shows the name of variables using the following codes: URB, urban; AGRI, agricultural, FOR, forest and semi-natural areas; WET, wetlands; WAT, waterbodies; ROAD, road density; RAIL, railways density. The squares below the diagonal show the bivariate plots and the squares above the diagonal the corresponding correlation coefficients, with level of significance indicated by symbols (.,*,**).

**
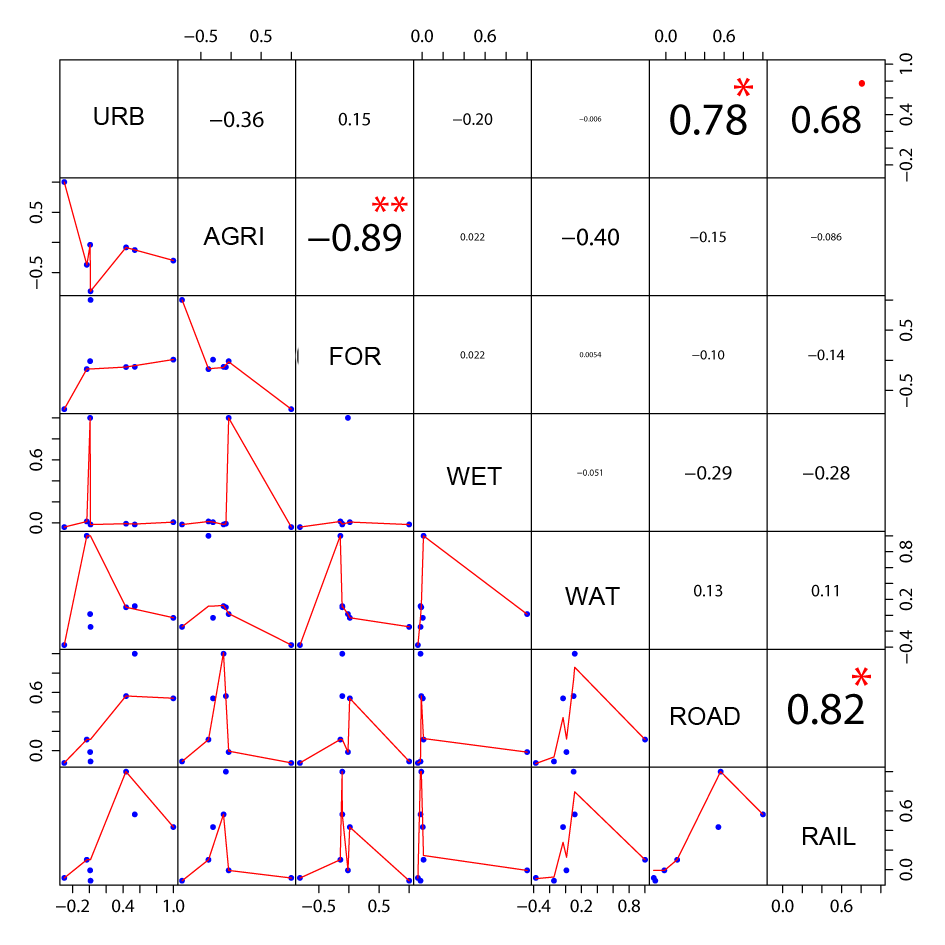
**
